# Data for distribution of various species of fecal coliforms in urban, rural and private drinking water sources in ten years period – A case study: Kermanshah, Iran

**DOI:** 10.1016/j.dib.2018.04.053

**Published:** 2018-04-23

**Authors:** Reza Davoodi, Meghdad Pirsaheb, Kamaladdin Karimyan, Vinod Kumar Gupta, Ali Reza Takhtshahi, Hooshmand Sharafi, Masoud Moradi

**Affiliations:** a Research Center for Environmental Determinants of Health, Kermanshah University of Medical Sciences, Kermanshah, Iran; b Environmental Health Research Center, Kurdistan University of Medical Sciences, Sanandaj, Iran; c Department of Environmental Health Engineering, School of Public Health, Tehran University of Medical Sciences, Tehran, Iran; d Department of Applied Chemistry, University of Johannesburg, Johannesburg, South Africa; e Water and Wastewater Company of Kermanshah Province, Kermanshah, Iran; f Student Research Committee, Kermanshah University of Medical Sciences, Kermanshah, Iran

**Keywords:** Fecal coliforms, Distribution, Drinking water sources, Kermanshah

## Abstract

This study was aimed to investigate the distribution of various species of fecal coliform in urban, rural and private drinking water sources of Kermanshah, in the west of Iran. For this study, data of ten years period (2006–2016) assessments of microbial quality regarding various species of *Fecal coliforms* was taken from health centers associated with urban, rural and private resources of Kermanshah city. A total number of 8643 samples were taken, 1851 samples from rural, 365 from urban and 4834 from private resources. The results showed that *Fecal coliforms*, *Escherichia coli* (*E. coli*) had the widest distribution in all urban, rural and private water resources (22.3%, 45.9% and 34%, respectively). Moreover, *E. coli* (47.5%) and *Klebsiella* (0.4%) had, respectively, the highest and lowest distribution in all months considered. Based on the results, *E.coli* exists mostly in water resources; it is therefore of particular importance in the monitoring of water resources.

## Specifications Table

**Table:** 

Subject area	Environmental Sciences
More specific subject area	Environmental Health
Type of data	Tables and figures
How data was acquired	For this study, data of the 10 years period (2006–2016) assessments of the microbial quality in terms of various species of fecal coliforms was taken from health centers associated with urban, rural and private sources of Kermanshah city.
Data format	Analyzed
Experimental factors	The presence of some coliforms indicate fecal contamination. The term "IMVIC" is an acronym for each of these tests. "I" is for indole test; "M" is for methyl red test; "V" is for Voges–Proskauer test, and "C" is for citrate test. The IMVIC test was not performed in about 3% of the all samples. Therefore, the results of these samples were not included in the study.
Experimental features	The above parameters that mentioned in abstract part, were analyzed according to the standards for water and wastewater treatment handbook.
Data source location	Kermanshah city, Iran
Data accessibility	Data are included in this article

## Value of the data

• Monitoring the quality of drinking water resources especially in term of microbial quality is necessary [1], [2], [3], [4], [5], [6], [7], due to the variety and wide range of drinking water sources in the Kermanshah city.
• The obtained data can assist in identifying contaminated resources and planning to adopt a long and short-term strategy for safe water supply.
• The obtained data revealed that rural water resources had more distribution of the various fecal coliform species particularly *E. coli* due to lack of water resources protection, poor sanitation, and improper disinfection and as well as more exposed to environmental pollutants.
• The data of present study show that *Enterobacter aerogenes, Enterobacter agglomerans*, and *Klebsiella* were more abundant species in the cold months, while *Citrobacter freundii, E. coli* and *Enterobacter cloacea* were more abundant species in the warm months.

## 1 Data

Table 1 shows the distribution of fecal coliforms in urban, rural and private water resources of Kermanshah city based on IMVIC test. *E. coli* (22.3%) and *Klebsiella* (2%) were the most and least bacteria existent in urban water resources, respectively. In rural water sources, *Escherichia coli* (45.9%) and *Enterobacter cloacea* (2.6%) and in private sources *E. coli* (34%) and *Klebsiella* (1.3%) had the most and least existent, respectively.

**Table 1:** Distribution of fecal coliforms based on different sources of drinking water.

**Bacteria**	**Distribution (%)**		
	**Urban sources**	**Rural sources**	**Private sources**
*Citrobacter freundii*	14	13.7	14.8
*Escherichia coli*	22.3	45.9	34
*Enterobacter aerogenes*	3.3	7.6	6.3
*Enterobacter agglomerans*	2.5	8.7	4.1
*Enterobacter cloacea*	4.3	2.6	2.5
*Klebsiella*	2	3.8	1.3
No *Fecal coliform*	51.6	17.7	37
Total	100	100	100
Number of samples	1851	4834	2001

Table 2, Table 3 present fecal coliforms distribution concerning months of the year and the amount of residual chlorine in water sources of the city. The results showed that *E. coli* with an average of 38.1% and *Klebsiella* with 2.8% had the highest and lowest distributions, respectively. The results also showed a significant decrease of distribution of fecal coliforms with increasing residual chlorine, while a decreasing trend is observed from the dose of 0.8 mg/L.

**Table 2:** Distribution of fecal coliform based on the months of year in drinking water of Kermanshah city.

**Seasons**	**Months**	**Number of samples**	**Distribution (%)**						
			***Citrobacter freundii***	***Escherichia coli***	***Enterobacter aerogenes***	***Enterobacter agglomerans***	***Enterobacter cloacea***	***Klebsiella***	**No*****Fecal coliform***
Spring	April	437	14.2	36.2	5.5	0	5.5	0.7	38
	May	805	10.8	41.7	5.7	2.5	0.6	4.3	24.3
	June	960	12.9	40.6	5.1	10.1	6.6	0.8	23.9
									
Summer	July	967	14.9	45	5.9	5.6	3.2	2.8	22.6
	August	1066	19.7	36.1	6	7.7	2.8	6.2	21.5
	September	834	17	47.5	7.6	0.9	2.8	0.4	23.9
									
Fall	October	761	13.6	33.4	8.8	7.7	2.8	0.4	28.8
	November	708	8.9	38.6	12.6	3.4	0.1	1.7	34.7
	December	666	13.7	31.8	2.1	15.5	0.2	0.5	36.3
									
Winter	January	553	4.7	31.5	4.9	11.9	6.3	4	36.7
	February	497	11.7	27.4	7.2	6	0.6	6.4	40.6
	March	433	7	37.9	3.9	4.4	1.6	0.9	44.3
								
Average		–	12.42	38.1	6.4	6.06	3	2.8	29.3

**Table 3:** Distribution of *Fecal coliforms* regarding the amount of residual chlorine in water resources of Kermanshah city.

**Residual chlorine range (mg/L)**	**Number of samples**	**Distribution (%)**							
		***Citrobacter freundii***	***Escherichia coli***	***Enterobacter aerogenes***	***Enterobacter agglomerans***	***Enterobacter cloacea***	***Klebsiella***	***No fecal coliform***	**Total**
0	5837	15.2	42.6	7.1	7.5	3.3	3.5	21.1	100
0.5–0	1376	13.5	34.6	5.9	5	3.1	2.2	37.6	100
0.5–0.8	821	11.4	26.8	5	4	2.5	1.8	46.8	100
More than 0.8	609	9	19	3	2	2.4	0.8	63.4	100
Average		11.27	38.1	6.4	4.62	3	2.8	29.1	100

## 2 Materials and methods

### 2.1 Study area

**Fig. 1:**
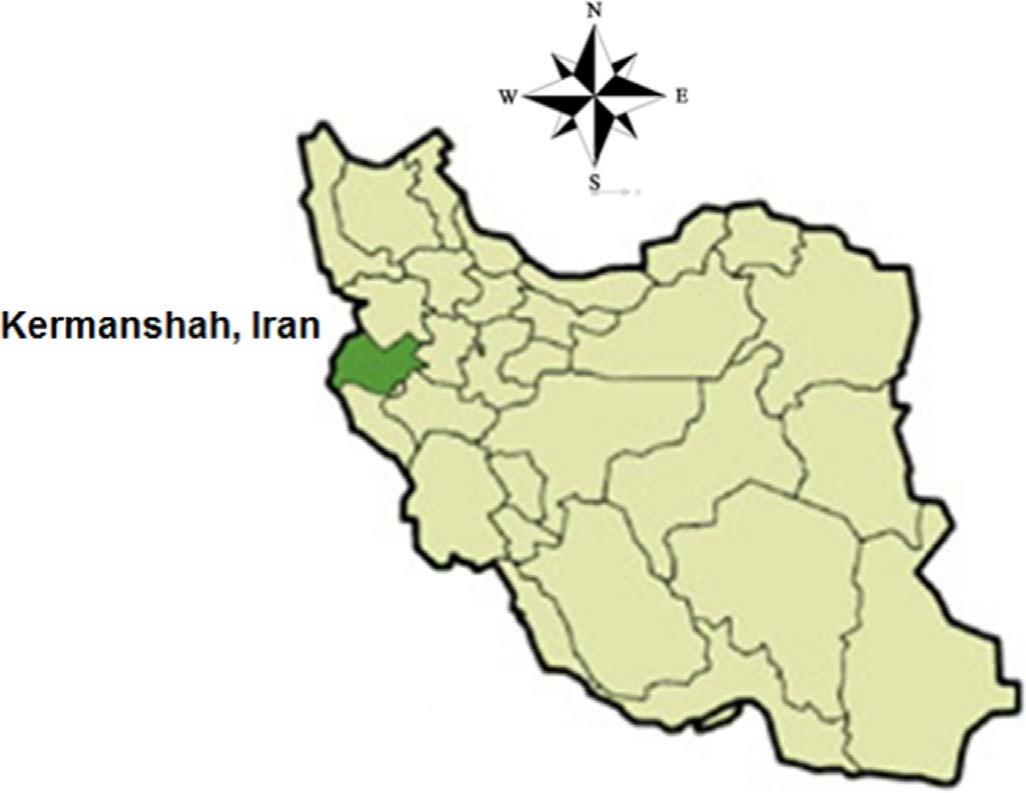
Map of the study area (Kermanshah city, Iran).

Kermanshah city with 24,500 m^2^ area and an altitude of 1200 m above sea level, locates in 47° and 4" east and 19° and 34" north in the west of Iran (Fig. 1). The city has a population over 1,945,227 people. Kermanshah has a population of more than a million people, that the main source of drinking water provided from underground water supplies [8], [9], [10], [11], [12], [13]. The above-mentioned water sources are threatened by various sources of pollution (especially urban, industrial and hospital wastewater) [14], [15], [16], [17], [18], [19].

### 2.2 Measurement and data collection

For this study, data of the ten years period (2006–2016) assessments of the microbial quality in terms of various species of *Fecal coliforms* was taken from health centers associated with urban, rural and private sources of Kermanshah city. Given that the study was conducted based on census, all results of measuring samples during the 10 years (8643 samples) were analyzed. The number of samples in rural, urban and private resources was 1851, 365 and 4834, respectively. The method to identify various species of fecal coliforms was according to standard methods [20], [21], [22], [23], [24], [25], [26], [27]. It is necessary to clarify that the IMVIC test (for determination of various species of fecal coliform), was not performed in about 3% of the all samples. Therefore, the results of these samples were not included in the study.
