# Supplementary material for: Data for distribution of various species of fecal coliforms in urban, rural and private drinking water sources in ten years period – A case study: Kermanshah, Iran
Source: Data Brief. 2018 Apr 23;18:1544–50. doi: 10.1016/j.dib.2018.04.053 (PMC5997978; doi:10.1016/j.dib.2018.04.053)
Supplement: Supplementary file 1 — Supplementary material [file mmc1.docx]

**Dear Editor-in-Chief**

Data In Brief

I would like to submit “**Distribution of various** **species of fecal coliform in urban, rural and private drinking water sources- Case study: Kermanshah, Iran”** as an original article to your prestigious journal.

All authors have read and approved the content of the manuscript and agreed with transfer of the copyright in case accepted for publication. The authors also certify that the manuscript has not been submitted or published elsewhere. All the authors contributed significantly to fit authorships criteria. No potential conflict of interest exists.

Sincerely Yours,

Corresponding Author

Dr.Masoud Moradi
